# Cleavage of Armadillo/beta-catenin by the caspase DrICE in *Drosophila *apoptotic epithelial cells

**DOI:** 10.1186/1471-213X-9-15

**Published:** 2009-02-20

**Authors:** Thomas Kessler, H Arno J Müller

**Affiliations:** 1 Division of Cell and Developmental Biology, College of Life Sciences, University of Dundee, Dundee, Scotland, UK; 2 Institut für Genetik, Heinrich Heine Universität, Düsseldorf, Germany; 3 Department of Vertebrate Genomics, Max Planck Institute for Molecular Genetics, Berlin, Germany

## Abstract

**Background:**

During apoptosis cells become profoundly restructured through concerted cleavage of cellular proteins by caspases. In epithelial tissues, apoptotic cells loose their apical/basal polarity and are extruded from the epithelium. We used the *Drosophila *embryo as a system to investigate the regulation of components of the zonula adherens during apoptosis. Since Armadillo/beta-catenin (Arm) is a major regulator of cadherin-mediated adhesion, we analyzed the mechanisms of Arm proteolysis in apoptosis.

**Results:**

We define early and late apoptotic stages and find that early in apoptosis *D*α-catenin remains relatively stable, while Arm and *D*E-cadherin protein levels are strongly reduced. Arm is cleaved by caspases in embryo extracts and we provide evidence that the caspase-3 homolog drICE cleaves Arm in vitro and in vivo. Cleavage by drICE creates a stable protein fragment that remains associated with the plasma membrane early in apoptosis. To further understand the role of caspase-mediated cleavage of Arm, we examined potential caspase cleavage sites and found that drICE cleaves Arm at a unique DQVD motif in the N-terminal domain of the protein. Mutation of the drICE cleavage site in Arm results in a protein that is not cleaved in vitro and in vivo. Furthermore we provide evidence that cleavage of Arm plays a role in the removal of *D*E-cadherin from the plasma membrane during apoptosis.

**Conclusion:**

This study defines the specificity of caspase cleavage of Arm in *Drosophila *apoptotic cells. Our data suggest that N-terminal truncation of Arm by caspases is evolutionarily conserved and thus might provide a principal mechanism involved in the disassembly of adherens junctions during apoptosis.

## Background

Apoptosis is accompanied by a stereotypical set of morphological events almost invariable from *C. elegans *to man [1]. The main features of the apoptotic cell phenotype are the condensation of the cytoplasm, breakdown of nuclear integrity, cell rounding, membrane blebbing, and in epithelial cells the loss of cell polarity and cell junctions [1-4]. These morphological changes are caused by proteolytic cleavage of key proteins by a group of aspartate-specific cystein proteases, called caspases [5-7]. Active caspases recognize and cleave their target proteins at defined tetra-peptide motifs; the cleavage site being located after an aspartate residue in the C-terminal most P1 position of a given tetra-peptide motif [8]. Cleavage of the substrates can lead to their inactivation or activation. For example, cleavage of ICAD (Inhibitor of caspase activated DNAse) releases the active CAD endonuclease, which triggers DNA fragmentation [9,10]. On the other hand, cleavage of Rho-associated Kinase-1 (ROCK-1) by caspase-3 leads to an activated form of the kinase that promotes membrane blebbing [2,3]. Although many caspase substrates have been identified by proteomic approaches [11], the overall mechanisms and cellular consequences of target cleavage remain to be elucidated.

Early during apoptosis in epithelia, cells lose contact and are extruded by the neighbouring cells in a active process [12]. While many cell adhesion molecules are cleaved by caspases in vitro, the relevance of such cleavage has not been addressed in vivo mostly due to the lack of a genetically tractable system. We have employed early *Drosophila *embryos as a model to follow the fate of the cadherin-catenin cell adhesion complex during apoptosis. The first epithelium formed in *Drosophila *embryogenesis is the blastoderm epithelium, a simple epithelial monolayer. A large part of the blastoderm epithelium matures gradually into the ectodermal epithelial sheet and the integrity of this epithelium depends on the activity of the cadherin-catenin system of cell adhesion molecules [13]. *D*E-cadherin, Arm (beta-catenin), and *Dα*-catenin accumulate in adherens junctions and form a typical apical belt-like structure [14,15]. Cadherins and catenins form stable Ca^2+^-dependent cell adhesion between epithelial cells and are instructive to organize the apical actin cytoskeleton [16,17]. *D*E-cadherin and Arm are essential to mediate cell adhesion during the establishment of cell junctions in early development [18-22].

The aim of this study was to examine the fate of the individual components of the cadherin-catenin system during apoptosis and to determine the functional relevance of their modification by caspases. The mutation *thread (th) *provides a good tool to study morphological changes in apoptotic cells, because the molecular mechanism of apoptosis induction in the absence of *th *has been well characterized [23-27]. *th *encodes for the cellular caspase inhibitor DIAP1, which is essential for the survival of all cells in the early embryo. Embryos homozygous for *th *loss of function alleles develop normally until mid-gastrulation, however shortly after this stage all cells undergo apoptosis because of unrestrained caspase activation [24,28,29]. One early response of apoptotic cells in DIAP1 null mutants is a change in cell shape associated with an apparent loss in epithelial polarity and cell adhesion [24]. We show that full length *D*E-cadherin and Arm are cleared from the membrane, while *Dα*-catenin is more stable during apoptosis. Arm is proteolytically cleaved by z-VAD-FMK sensitive proteases present in embryo extract from apoptotic cells. We present evidence that Arm is cleaved by the caspase-3 homolog drICE, and determined a DQVD motif as specific caspase cleavage site within the N-terminus of Arm. Our results show that cleavage of Arm results in a truncated C-terminal Arm cleavage product, Arm^ΔN ^that maintains its localization with *Dα*-catenin even after the onset of apoptotic responses. Cleavage of Arm can be prevented in apoptotic cells by a single amino acid exchange in the N-terminal DQVD motif. Expression of caspase-resistant Arm in the embryo leads to a delay of the removal of *D*E-cadherin from the plasma membrane of apoptotic cells. These data suggested that drICE-specific cleavage of Arm is responsible for the loss of *D*E-cadherin from cell junctions and thus might contribute to the degeneration of epithelial integrity during apoptosis. However we find that Arm^ΔN ^is still able to mediate cell adhesion in an *arm *mutant background. Therefore caspase cleavage of Arm does not render Arm non-functional suggesting that in addition to Arm cleavage other modification of the protein complex is required for the down-regulation of cadherin-dependent cell adhesion in apoptotic epithelial cells.

## Results

### Arm is degraded in apoptotic cells

**Figure 1:**
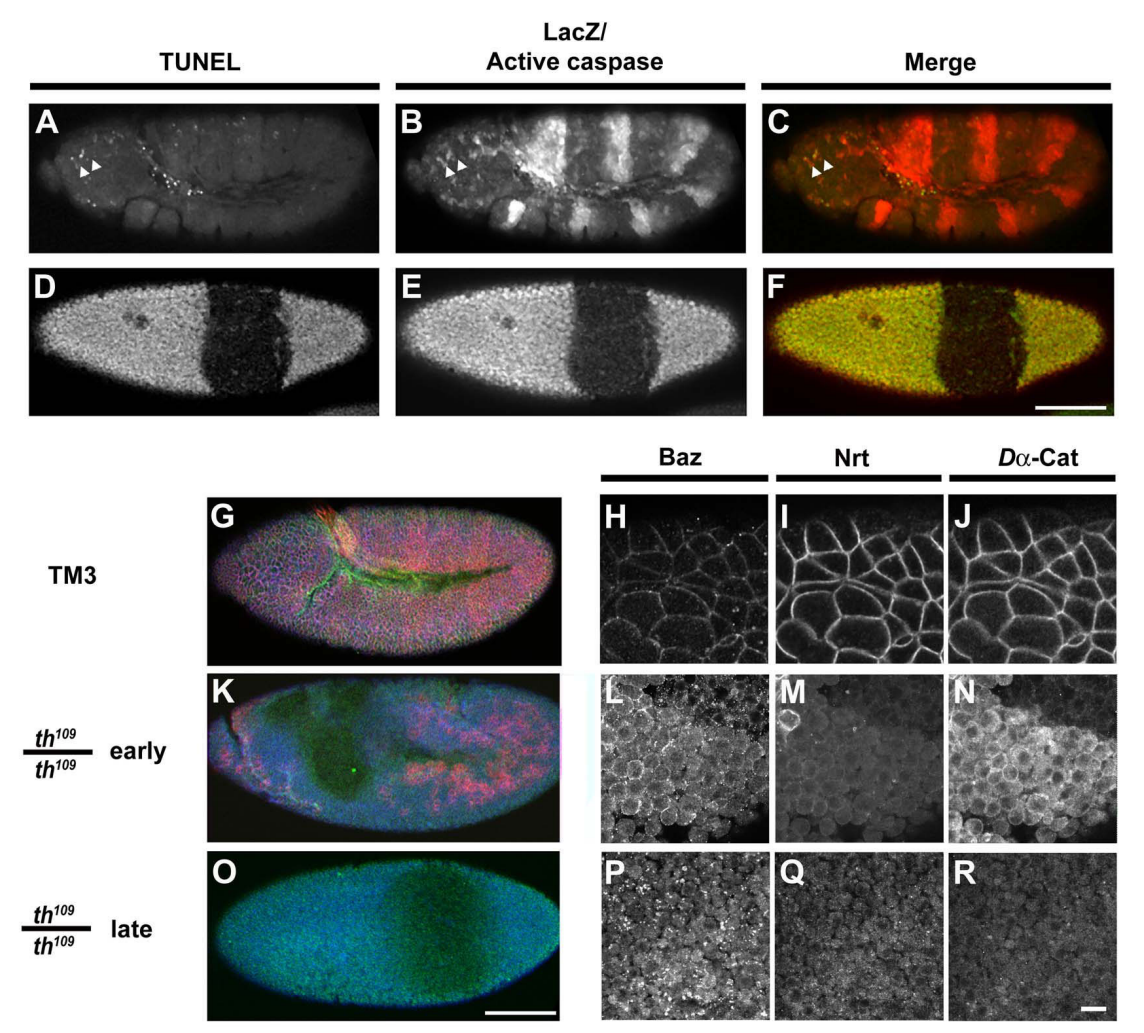
**DIAP1 loss of function mutation results in altered cell polarity and adhesion**. (A-F) Apoptosis in *th*^109 ^heterozygous and homozygous embryos; (A-C) *th*^109 ^heterozygous embryo stained for fragmented DNA (TUNEL) (A), activation of the executioner caspase drICE (B) and expression of *ftz::lacZ *(B); the *ftz::lacZ *transgene results in a striped expression pattern of beta-galactosidase (beta-gal) and indicates a copy of the wild type allele of the DIAP1 gene. Arrowheads in (A-C) indicate apoptotic cells in the head region. (D-F) *th*^109 ^homozygous embryo stained for fragmented DNA (D) and expression of *ftz::lacZ *and active drICE (E). (C, F) Merged images show anti beta-galactosidase and anti active-drICE staining in red and TUNEL signal in green/yellow. (G-R) *th*^109 ^mutant embryos stained for Bazooka (Baz, in blue) (H, L, P), Neurotactin (Nrt, red) (I, M, Q) and *Dα*-Catenin (*Dα*-Cat, green) (J, N, R). (G-J) *th*^109 ^heterozygous embryo; (K-N) *th*^109 ^homozygous embryo 45 min after cephalic furrow formation. (O-R) *th*^109 ^homozygous embryo 60 min after cephalic furrow formation. Scale bars in (F) 40 μm valid for (A-F, G, K, O); scale bar in (R) 10 μm for (H-J, L-N, P-R).

The *Drosophila *cellular blastoderm embryo is composed of a simple epithelial monolayer covering a central yolk cell. The blastoderm epithelium undergoes a variety of morphogenetic movements during gastrulation including ventral furrow formation and germ band extension. To study the effects of caspase activation on these primary epithelial cells, we examined the components of the cadherin/catenin adhesion system in apoptotic cells using embryos homozygous for the DIAP1 loss-of-function mutation *th*^109^. We have shown before that DIAP1 mutant embryos undergo a complete and sudden block of all morphogenetic movements at the onset of germ band extension [24]. All cells in DIAP1mutant embryos exhibit typical features of apoptosis, such as DNA fragmentation, caspase activation, cell rounding and membrane blebbing (Fig. 1A–F) [24-26,28]. Unlike in wildtype embryos where apoptosis is not detectable during gastrulation, in gastrulating DIAP1 mutant embryos all cells become TUNEL-positive and accumulate high levels of processed caspase indicating that all cells have undergone apoptosis (Fig. 1D, E, F).

To examine the effects in DIAP1 mutant embryos on membrane polarity within the epithelium, we immunolocalized Bazooka (Baz), a component of the subapical region, *Dα*-catenin, a component of adherens junctions, and Neurotactin (Nrt), a marker of the basolateral membrane domains (Fig. 1G–R). We discriminate between two distinct developmental time points in DIAP1 mutant embryos: (1) The early stage, that lasts about 30 min after the first signs of the morphogenetic block (cephalic furrow formation + 45 min, represents developmental stage 8) and (2) The late stage, an hour after the morphogenetic block occurred (cephalic furrow formation + 60 min, corresponds to developmental stage 9). In wildtype or *th*^109 ^heterozygotes, these markers label the cells circumference at different levels along the apical to basal axis (Fig. 1G, H, I, J). In DIAP1 mutants, the distribution of the marker proteins is strongly impaired. In early stages of apoptosis, Baz and *Dα*-catenin are still detected at the cell surface, albeit at lower levels (Fig. 1K, L, N). Nrt can no longer be detected at the cell surface, suggesting that full-length Nrt is rapidly degraded during apoptosis and thus represents an early marker for apoptotic cells in the *Drosophila *embryo (Fig. 1M). In late stages of apoptosis, cells in DIAP1 mutants show low levels for all three markers (Fig. 1O, P, Q, R). Only Baz immunoreactivity can still be detected in a punctate staining pattern (Fig. 1P). We conclude that components of the subapical region and the adherens junctions become redistributed and presumably degraded during apoptosis.

**Figure 2:**
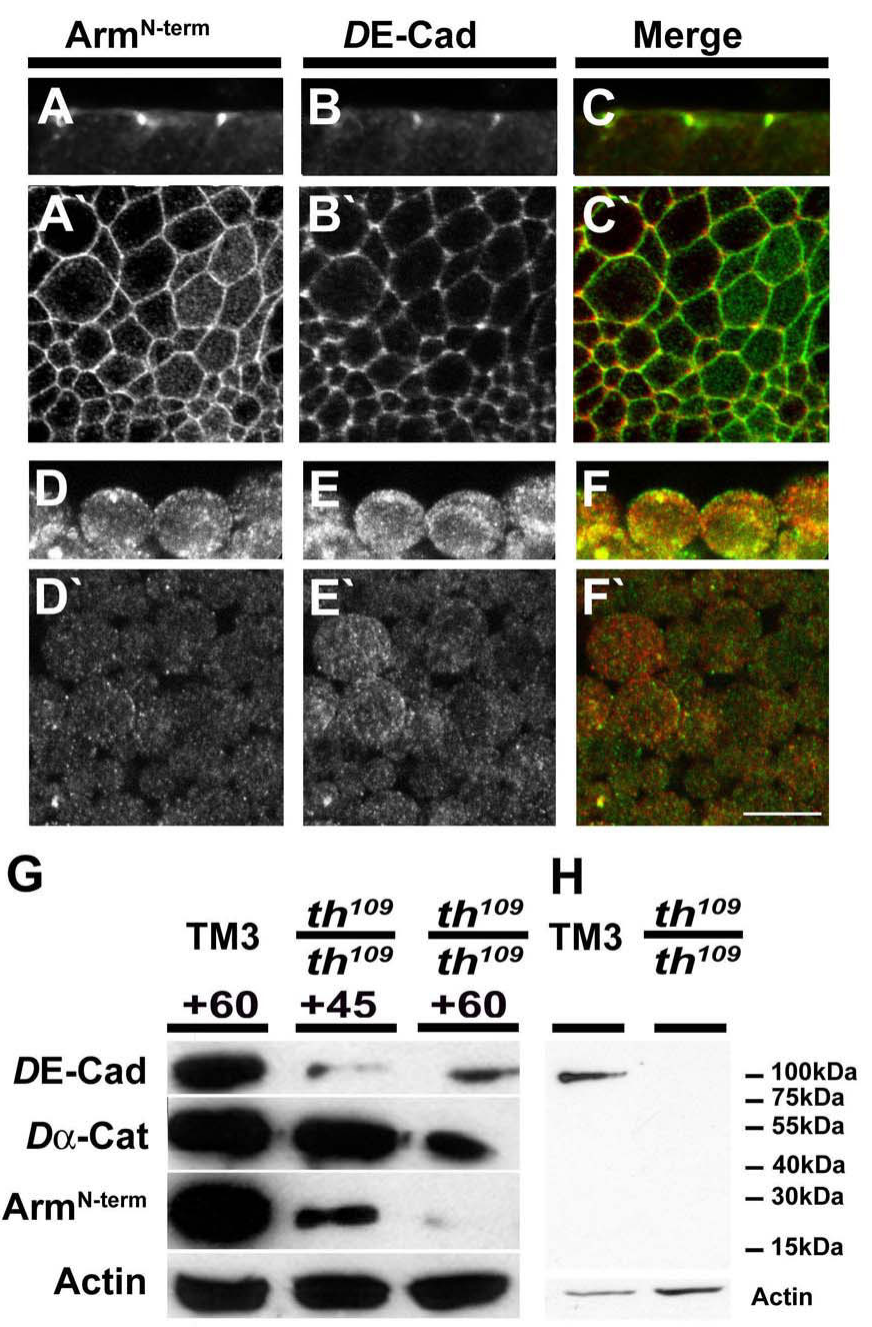
**Arm is degraded during apoptosis**. (A-C') *th*^109 ^heterozygous embryo (stage 9, i.e. 60 min after the onset of the morphogenetic arrest in *th*^109 ^homozygotes), stained for Arm (A, A') and *D*E-cadherin (B, B'). (A, B) confocal cross-section; surface view (A', B'). (D-F') Same stage *th*^109^homozygous embryo stained for Arm (D, D') and *D*E-cadherin (E, E'). (D, E) confocal cross section and surface view(D', E'). (C, C', F, F') Merged images with Arm in green and *D*E-cadherin in red. Scale bar in (F') for (A-F) represents 10 μm. (G) Western blot probing protein levels of *D*E-cadherin, *Dα*-Cat, Arm and Actin in protein extracts of *th*^109 ^heterozygous (wt) and *th*^109^homozygous embryos 45 min and 60 min after cephalic furrow formation (indicated as +45 and +60, respectively). (H) Western blot using anti-Arm^Nterm ^antibody to probe extracts of balancer controls (TM3) and *th*^109 ^homozygous embryos at time points 60 min after cephalic furrow formation; note that anti-Arm^Nterm ^antibody recognizes full length Arm protein in controls, but no degradation products in *th*^109 ^homozygotes. Actin was used as a loading control, because we found that Actin is not apparently cleaved in *th*^109 ^homozygous embryos at the time points indicated.

To gain a better picture of the redistribution and disassembly of adherens junctions during apoptosis, we took a closer look at the distribution and the protein levels of *D*E-cadherin and Arm in DIAP1 mutant embryos (Fig. 2). In wildtype and *th*^109 ^heterozygotes, *D*E-cadherin and Arm co-localise in apical adherens junctions, the zonula adherens (ZA) (Fig. 2A–C). At the late stage of apoptosis (>60 min after the onset of the morphogenetic arrest), cells have rounded up and exhibit only low levels of Arm protein (Fig. 2D, D'). Similarly, *D*E-cadherin is strongly reduced and is predominantly found in the cytoplasm (Fig. 2E, E', F, F'). The change in the subcellular localization of junctional proteins in apoptotic cells parallels the changes in overall levels of the full-length proteins. In the early apoptotic stage the levels of full-length *D*E-cadherin and Arm protein are strongly reduced, while *Dα*-catenin levels remain largely unimpaired (Fig. 2G). In the late stage, both Arm and *Dα*-catenin amounts are further reduced. Indeed, the full-length Arm protein is at the limit of detection in late apoptotic embryos suggesting that Arm is efficiently degraded during apoptosis (Fig. 2H). Remarkably, *Dα*-catenin appears very stable in early apoptotic cells; this result is consistent with the persistent membrane localization of *Dα*-catenin in the early stage of apoptosis (Fig. 1N). We conclude that during apoptosis the components of the cadherin/catenin system are progressively degraded and eventually redistributed from the plasma membrane to the cytoplasm.

### Arm is degraded by zVAD-sensitive proteases in extracts of apoptotic cells

Our observation on the major adherens junction proteins during apoptosis of DIAP1 mutant cells showed that Arm distribution and protein levels are strongly affected. Arm is quickly degraded at the time when caspases become activated. To test whether Arm is degraded by caspase activity in apoptotic cells, we reconstituted cleavage of Arm in vitro using an embryo extract system.

**Figure 3:**
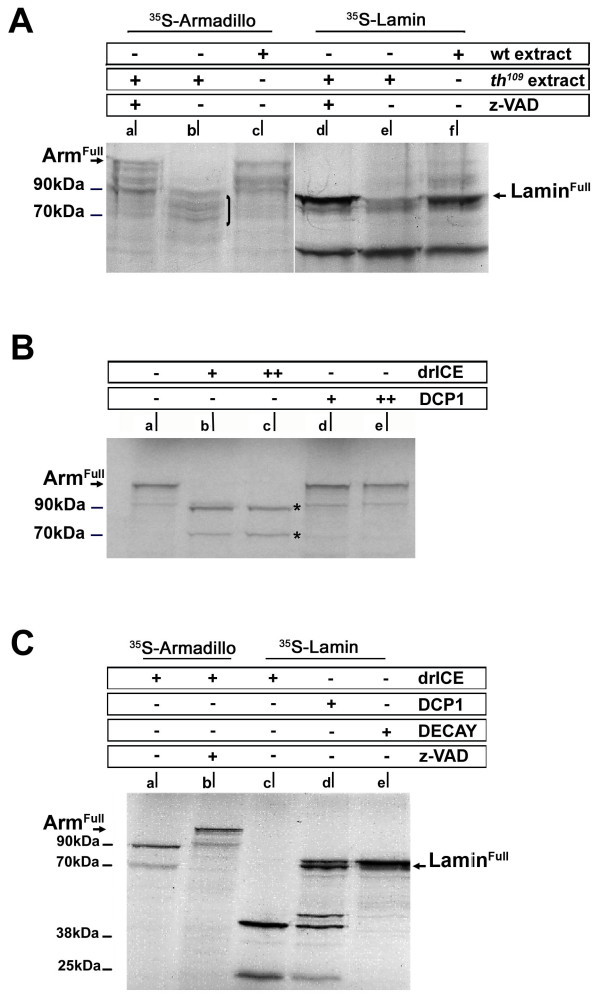
**Arm is cleaved by caspases in vitro**. (A) Cleavage of Arm and Lamin Dmo by embryonic extracts in vitro. ^35^S-labeled Arm (lane a-c) or Lamin Dmo (d-f) was incubated with protein extracts of wildtype embryos (wt extract; c, f) or extracts prepared from embryos obtained from *th*^109 ^heterozygous flies (1/4 of the embryos are homozygous for *th*^109 ^indicated as *th*^109 ^extract; b, e). The *th*^109 ^extract contains proteolytic activity that leads to quantitative degradation of Lamin Dmo (e) and Arm (b). ^35^S-labeled Arm appears as a range of bands suggesting premature termination during in vitro translation. Arm cleavage results in proteolytic fragments with the apparent M_r _between 90 kDa and 70 kDa suggesting N-terminal cleavage of all isoforms produced in vitro (bracket in b). Addition of the pan-caspase inhibitor z-VAD-FMK completely prevents degradation of Arm (a) and Lamin Dmo (d). (B) In vitro cleavage of Arm by recombinant drICE, but not DCP-1. ^35^S-labeled Arm was incubated with 0.5 μg and 1 μg recombinant drICE (b+c) or DCP1 (d, e), respectively. drICE cleavage of Arm leads to stable proteolytic products with the M_r _of 90 kDa and 70 kDa, the 70 kDa product again likely represents a cleaved isoform produced by premature termination of the substrate (lower stars in b and c). (C) drICE and DCP1 are both active in vitro. (a) In vitro translated, S^35^-methionine labelled Arm is cleaved by drICE resulting into the 90 kDa Arm cleavage product. (b) Cleavage of S^35^-methionine labelled Arm can be prevented by addition of z-VAD-FMK to the lysate prior to incubation with drICE. (c) S^35^-methionine labelled Lamin Dmo is cleaved by drICE into 20 kDa and 50 kDa fragments (d) DCP1 cleaves Lamin Dmo into 20 kDa, 50 kDa and 55 kDa fragments. Another caspase3 (and -7) related caspase Decay [55] is unable to cleave Lamin Dmo (lane e).

Extracts of wildtype or DIAP1 mutant embryos where made under conditions that keep caspases active, while other proteolytic activity was blocked [24]. To control the activity of the extracts, we tested nuclear Lamin Dmo as a well-characterized substrate of caspases (Fig. 3A, lanes d-f; [30]). ^35^S-methionine labelled Lamin Dmo was produced in reticulocyte lysate and added to the embryo extracts. ^35^S-Lamin Dmo was readily cleaved by extracts from DIAP1 mutants, while extracts of wildtype embryos had no effect on the stability of ^35^S-Lamin Dmo (Fig. 3A, lanes e, f). To determine whether the observed cleavage of ^35^S-Lamin Dmo was based upon caspase activity, we employed the pan-caspase inhibitor zVAD-FMK [31]. When zVAD-FMK was added to the extract prior to incubation with ^35^S-Lamin Dmo, cleavage of Lamin was completely abolished (Fig. 3A, lane d). Thus *Drosophila *embryo extracts provide a simple system to test caspase activity towards selected substrates in vitro.

We next tested the capability of embryo extracts to cleave ^35^S-Arm (Fig. 3A, lanes a-c). In vitro translated ^35^S-Arm was stable when added to extracts from wild-type embryos, but incubation with extracts from DIAP1 mutant embryos lead to cleavage of ^35^S-Arm and the formation of stable fragments at apparent M_r _range from 90 kDa to 70 kDa (Fig. 3A, lanes b, c). Both, the initial ^35^S-Arm substrate and the cleavage product were found to represent several bands, which were likely to be produced by premature termination during the in vitro translation reaction. Again, cleavage was based on zVAD-sensitive protease activity in the extract (Fig. 3A, lane a). We conclude that Arm is cleaved in vitro by a zVAD-sensitive protease, most likely reflecting caspase activity, which is present in extracts from DIAP1 mutant embryos, but not in extracts made from wildtype embryos. These data suggest that Arm is cleaved in apoptotic cells and that this cleavage might produce a stable proteolytic fragment that might persist throughout late stages of apoptosis.

### Arm is cleaved by the caspase drICE at the DQVD^88 ^motif

The results from the embryo lysate system suggested that during apoptosis Arm is cleaved by caspases, because the proteolytic activity in the embryo extracts was completely inhibited by the caspase inhibitor zVAD-FMK. We therefore sought to determine which of the *Drosophila *caspases cleaves Arm.

It had been shown before that human beta-catenin is a target of caspase-3 mediated cleavage in cultured cells [32]. To determine which of the *Drosophila *effector caspase cleaves Arm, we performed experiments with recombinant, bacterially produced caspase-3 homologues drICE and DCP1. ^35^S-Arm was incubated with different amounts of caspases and the stability of Arm protein was determined by SDS-PAGE and autoradiography (Fig. 3B). drICE was able to cleave Arm into a stable 90 kDa fragment while DCP1 did not cleave Arm under these conditions (Fig. 3B). Cleavage of ^35^S-Arm by drICE was completely blocked by addition of zVAD-FMK to the assay (Fig. 3C, a,b). We conclude that Arm is cleaved by drICE releasing a stable protein fragment, which corresponds in size to the fragment produced in the embryo lysate assay. These results suggest that the activity of the apoptotic embryo extract, which cleaves Arm, might represent endogenous drICE activity (see below). As control for the activities of the recombinant caspases, we tested their capability to cleave the known target Lamin Dmo [30]. Both drICE and DCP1 were able to cleave ^35^S-Lamin Dmo, although the pattern of Lamin cleavage produced by the two caspases was distinct (Fig. 3C, c, d). As a further control, full length ^35^S-Lamin Dmo remained uncleaved in the presence of the caspase Decay under the same conditions (Fig. 3C).

**Figure 4:**
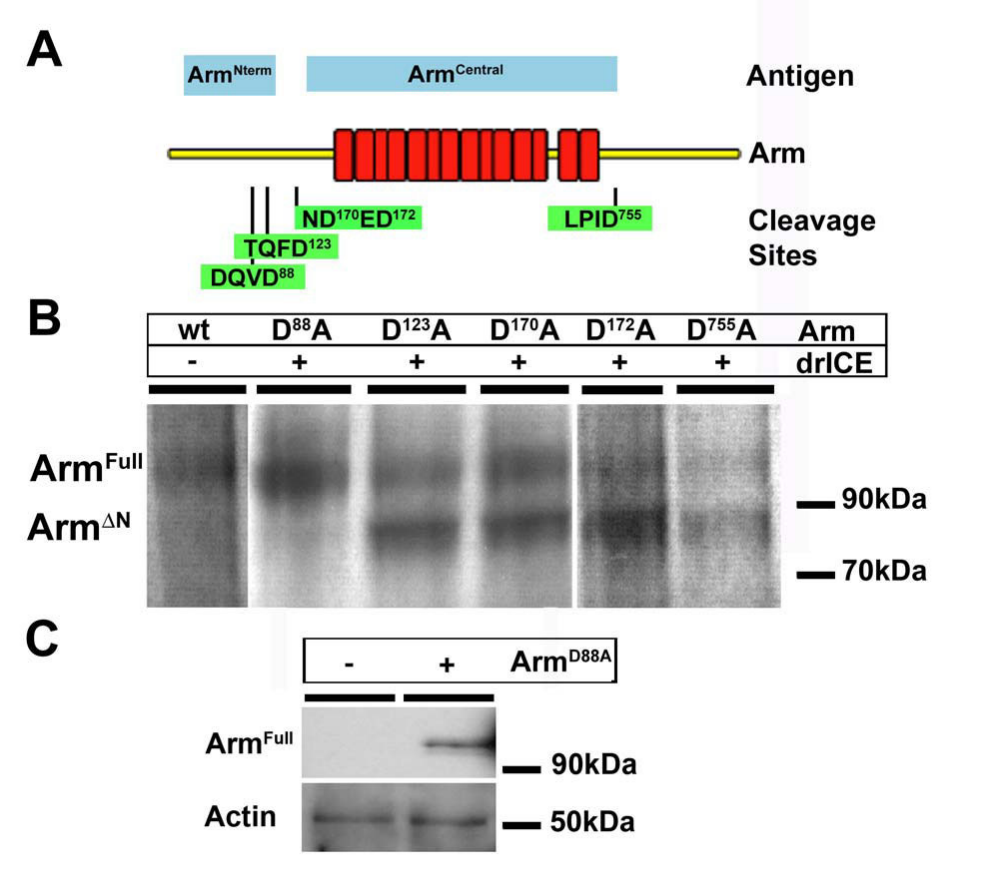
**The N-terminus of Arm is cleaved by drICE**. (A) Schematic representation of the domain structure of Arm. Arm repeats are indicated as red boxes. Putative caspase cleavage sites are indicated in green boxes. Blue bars depict the extent of the antigens used to produce Arm^Nterm ^(monoclonal antibody 7A1) and Arm^Central^, respectively. (B) Cleavage of in vitro translated, ^35^S-methionine labelled mutated forms of Arm. As control full length Arm was incubated with buffer (control). Mutated variants of Arm (D^88^A, D^123^A, D^170^A, D^172^A and D^755^A) were incubated with recombinant drICE. Upon incubation with drICE stable products were identified of all of the Arm mutants except for D^88^A. The cleavage products have a M_r _of approximately 90 kDa and are marked as Arm^ΔN^. Full length Arm (Arm^Full^, M_r _110 kDa) is reduced except for D^88^A, which remains stable. (C) Western blot of embryo extracts of *th*^109 ^homozygous embryos (- Arm^D88A^) and *th*^109 ^homozygous embryos expressing Arm^D88A ^from a transgene (*+ *Arm^D88A^); expression of Arm^D88A ^was achieved using the Gal4/UAS system with Gal4 expressed under the control of a maternal beta-tubulin promoter (*mat15::Gal4*). Hand selected embryos of either genotype were subjected to Western blotting using Arm^Nterm ^antibody; the blot was stripped and re-probed using an anti-Actin antibody for loading control; note that Actin remains stable in *th*^109 ^apoptotic cells.

After we determined that drICE cleaves Arm, we next asked at which sites Arm was cleaved in apoptotic cells. Several tetra-peptide motifs of human beta-Catenin can be cleaved by caspase-3 after induction of apoptosis [4,32]. From the various motifs found in human beta-Catenin, only one motif is conserved in Arm: a TQFD motif at amino acid position 119–123 (TQFD^123^, Fig. 4A). In addition, we detected the potentially highly specific caspase cleavage motif DQVD at amino acids 84–88 within the N-terminal sequence of Arm.

Caspases are strictly dependent on Asp in the P1 position of target sequences and changing P1 from Asp to Ala prevents cleavage [8]. The substrate specificities of DCP-1 and drICE are very similar, with a specificity for DXVD peptide motifs, where X has a preference for E, D, Q, T, V or A [33]. To determine which of the five potential caspase cleavage motifs in Arm was used by drICE, we performed an in vitro mutagenesis experiment. The Arm cDNA sequence encoding putative caspase cleavage motifs was altered to create missense mutations changing Asp to Ala at P1; such mutations render these cleavage motifs non-functional. We mutated a putative cleavage site just N-terminal to the Arm repeats (ArmD170A and ArmD172A), a region that was shown to be crucial for *Dα*-catenin binding [34,35]. Another potential motif C-terminal to the *D*E-cadherin binding region was also mutated (ArmD755A; Fig. 4A). Furthermore, we mutated the cleavage sites DQVD^88 ^(ArmD88A) and TQFD^123 ^(ArmD123A) in the N-terminus of Arm. The various mutated forms of Arm were radio-labelled and subjected to drICE cleavage in vitro. Mutations of the non-conserved motifs had no inhibitory effect on the cleavage and always lead to stable 90 kDa products as seen with wildtype Arm (Fig. 4B). Even mutation of the conserved TQFD^123 ^motif did not prevent cleavage by drICE (Fig. 4B). However cleavage was completely abolished when the DQVD^88 ^motif was altered by the mutation D88A (Fig. 4B).

To test whether Arm is indeed cleaved at DQVD^88 ^in the embryo, we generated transgenic flies that allow conditional expression of the mutated drICE-resistant variant of Arm, Arm^D88A^. When analyzed by Western blotting, Arm^D88A ^remains intact in late stage apoptotic cells (Fig. 4C). These results indicate that Arm is cleaved in the N-terminal domain during apoptosis. Furthermore, cleavage of Arm in apoptotic cells can be prevented by mutation of a single caspase consensus site in the N-terminal half of the protein.

### Cleaved Arm remains associated with the cell surface in apoptotic cells

The in vitro data in combination with the over-expression data indicated that drICE-mediated removal of the N-terminal 88 amino acids leads to a stable cleavage product at a size of approximately 90 kDa. This result is in contrast to the findings described in the beginning where we found a strong decrease of Arm levels in DIAP1 mutant cells. However, since the commonly used monoclonal antibody 7A1 is directed against the N-terminus of the protein, cleavage at DQVD^88 ^is predicted to remove the epitope for this antibody [36,37]. Thus the observed decrease in the level of full length Arm in DIAP1 mutant cells might reflect the removal of the epitope of the anti-Arm^Nterm ^antibody rather than a decrease in the total amount of Arm protein.

**Figure 5:**
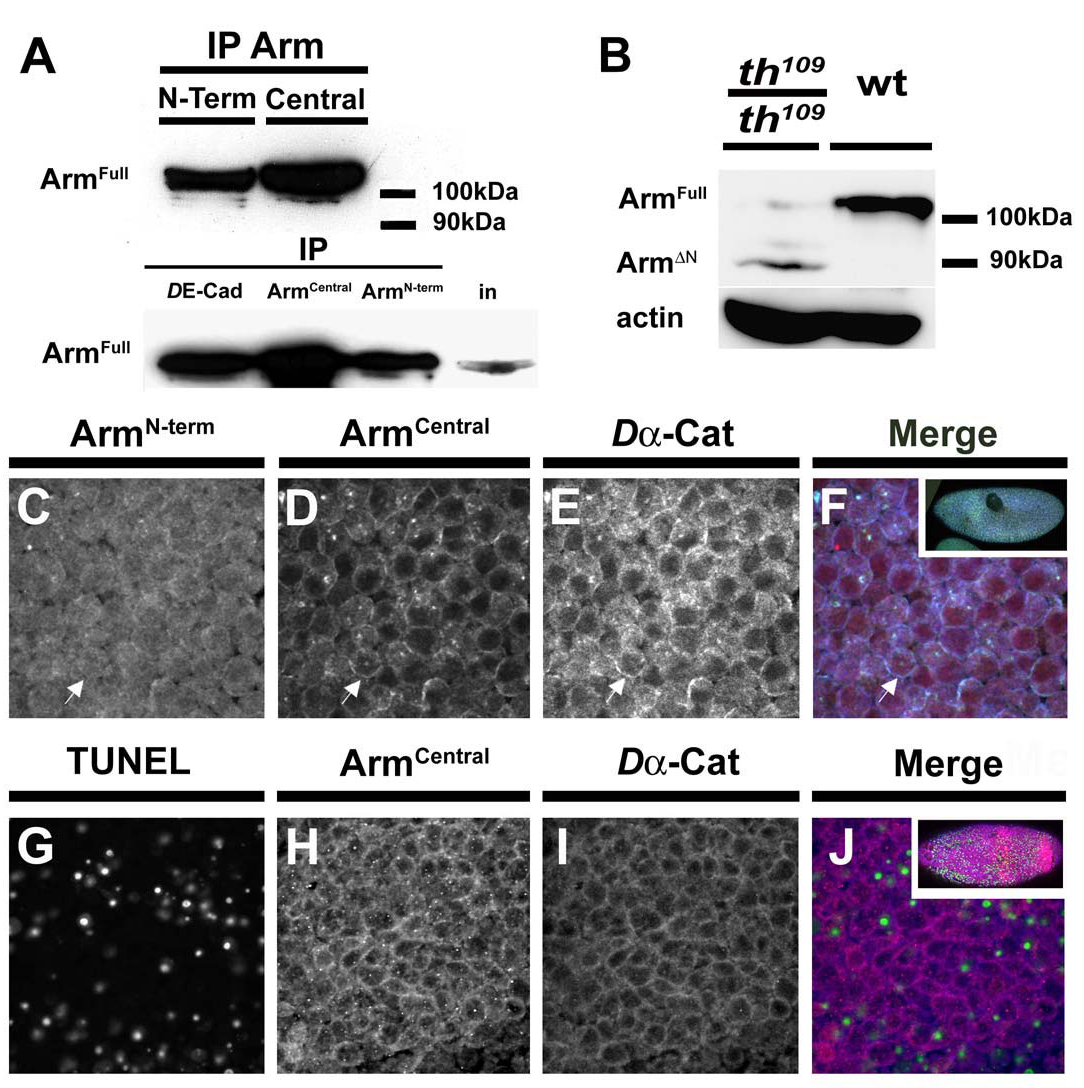
**A stable Arm cleavage product persists in apoptotic cells**. (A) Arm^Central ^antibody immunoprecipitates full length Arm; Full length Arm (M_r _110 kDa; indicated as Arm^Full^) is immunoprecipitated (IP) by the Arm^Nterm ^or the Arm^Central ^antibody. In Western blots of these immunoprecipitates Arm^Full ^is detected by the Arm^Nterm ^antibody. *D*E-cadherin or Arm antibodies were used to immunoprecipitate proteins from embryo extracts (IP) as indicated. Immunoprecipitates were then subjected to Western blotting using Arm^Central ^antiserum. Anti Arm^Central ^detects full length Arm in the input lane (in) and in immunoprecipitates by all three antibodies. (B) Detection of Arm^ΔN ^in protein extracts of *th*^109 ^homozygous embryos. Western blot of protein extracts from *th*^109 ^heterozygous (wt) and *th*^109 ^homozygous embryos. Levels of Arm^Full ^are decreased in *th*^109 ^homozygous embryos and a short form of Arm with M_r _~90 kDa is detected corresponding to Arm^ΔN^. Actin was used as loading control. (C-F) Localization of Arm^ΔN ^in *th*^109 ^homozygous embryos (45 min after onset of morphogenesis arrest) stained for Arm^Nterm ^(C), Arm^Central ^(D) and *Dα*-Cat (E). Arrows in (C-E) point to an apoptotic cell where full length Arm is absent from the plasma membrane but Arm^Central ^is still present (F) Merged image with Arm^Nterm ^in green, Arm^Central ^in red and *Dα*-Cat in blue. (G-J) *th*^109 ^homozygous embryo (60 min after onset of morphogenesis arrest) (inset in J) stained for (G) TUNEL, (H) Arm^Central ^and (I) *Dα*-Cat; (J) Overlay with TUNEL in green, Arm^Central ^in red and *Dα*-Cat in blue.

Because the specificity of the anti-Arm^Nterm ^antibody might prevent detection of an N-terminally truncated Arm cleavage product, we raised a polyclonal antibody against the central domain of Arm (amino acids 172–755) referred to as anti-Arm^Central ^antibody (Fig. 4A). The specificity of the anti-Arm^Central ^antibody was determined by immunofluorescence as well as by immunoprecipitation experiments (Fig. 5). In early embryos, anti-Arm^Central ^immunoreactivity always co-localised with the staining obtained with anti-Arm^Nterm ^(data not shown). Furthermore, anti-Arm^Central ^detects full length Arm, which was immunoprecipitated either with anti-Arm^Central^, anti-Arm^Nterm^, or anti-*D*E-cadherin antibodies (Fig. 5A). We conclude that the anti-Arm^Central ^antibody recognizes full length Arm protein in *Drosophila *embryos.

The anti-Arm^Central ^antibody allowed us to determine whether a stable Arm cleavage product was generated in the embryo. We performed a Western analysis of extracts obtained from hand-selected DIAP1 mutant embryos at the late apoptotic stage. While in wildtype embryos only full-length Arm can be detected, additional forms of the protein appear in DIAP1 mutantembryos; the most prominent form with an apparent molecular weight of around 90 kDa (Fig. 5B). The 90 kDa form of Arm is stable as it persists until the late stage of apoptosis, at time points when full length Arm is no longer detectable (Fig. 5C, D, H). This experiment demonstrates the presence of a cleavage product of Arm at around 90 kDa in apoptotic cells. Strikingly, the 90 kDa fragment is not recognized by the anti Arm^Nterm ^antibody in extracts of apoptotic cells (Fig. 2H). Together these results strongly suggest that the 90 kDa fragment might indeed represent the N-terminal Arm fragment produced by drICE.

To determine the subcellular localization of the 90 kDa Arm fragment, DIAP1 mutant cells were co-stained with anti-Arm^Nterm^, anti-Arm^Central ^and anti *Dα*-catenin antibodies (Fig. 5C–J). While in early DIAP1 mutant embryos full-length Arm is not associated with the cell surface anymore, the anti-Arm^Central ^antibody shows a clear localization along the periphery of the cells (Fig. 5C, D). Interestingly, the staining of the anti-Arm^Central ^antibody co-localised with *Dα*-catenin suggesting that the cleaved Arm fragment is still associated with *Dα*-catenin (Fig. 5D–F). Even in cells that are TUNEL positive, Arm^Central ^and *Dα*-catenin still colocalise (Fig. 5G–J). These results demonstrate the generation of a stable 90 kDa central fragment of Arm, which maintains its localization at the cell periphery and colocalises with *Dα*-catenin during early stages of apoptosis.

### Cleavage of Arm by drICE is required for clearing of DE-cadherin from the cell membrane during apoptosis

**Figure 6:**
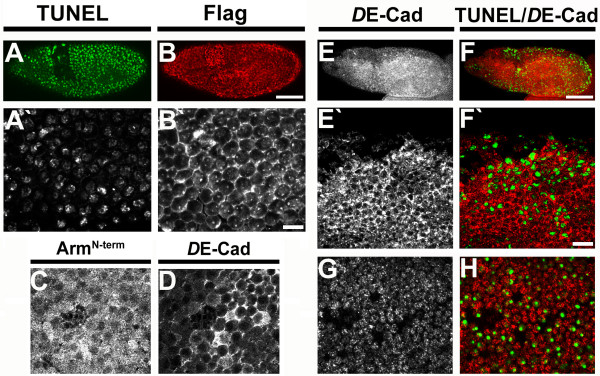
**Expression of Arm^D88A ^interferes with redistribution of *D*E-cadherin during apoptosis**. (A, B) *th*^109 ^homozygous embryo (stage 9, 60 min post morphogenetic block) expressing Arm^D88A ^stained for TUNEL (A, A') and expression of the Flag-tagged Arm^D88A ^transgene (B, B' anti-Flag). (C, D) *th*^109 ^homozygous embryo (60 min post cephalic furrow formation) expressing Arm^D88A ^and stained for Arm (C) and *D*E-cadherin (D); note persistent localization of *D*E-cadherin at the plasma membrane. (E-H) *D*E-cadherin localization in *th*^109 ^homozygous embryos with or without expression of Arm^D88A^. (E, F) *th*^109 ^homozygous embryo (60 min post cephalic furrow formation) expressing Arm^D88A ^stained for *D*E-cadherin (E, E'; red in F, F') and TUNEL (green in F, F'). (G, H) *th*^109 ^homozygous embryo (60 min post cephalic furrow formation) stained for *D*E-cadherin (red in H) and TUNEL (green in H). Scale bar in (B) and (F) 40 μm, scale bar in B') and F') 10 μm.

Our results showed that in apoptotic cells Arm is cleaved by the caspase drICE and that this cleavage generates a stable fragment of Arm. Upon over-expression of Arm^D88A ^in DIAP1 mutant embryos the execution of apoptosis occurred slightly delayed, but remained otherwise unimpaired. For example, DIAP1 mutant embryos expressing Arm^D88A ^show massive appearance of TUNEL positive cells in stage 9 similar to DIAP1 mutant embryos without Arm^D88A ^transgene expression (Fig. 6A, A'). While endogenous full length Arm protein is no longer associated with the surface of apoptotic cells, the Flag-tagged Arm^D88A ^transgenic protein remains associated with the plasma membrane of late apoptotic cells (Fig. 6B, B'). This result suggests that cleavage of Arm at DQVD^88 ^is required for removal of membrane associated full length Arm in apoptosis. Expression of Arm^D88A ^also resulted in a more persistent localisation of *D*E-cadherin at the membrane in a phase during apoptosis when it is normally redistributed to the cytoplasm (Fig. 6C–H). This result indicates that drICE-mediated cleavage of Arm at the N-terminal domain is required for the observed redistribution of *D*E-cadherin and might contribute to the breakdown of cell-cell adhesion in apoptosis.

**Figure 7:**
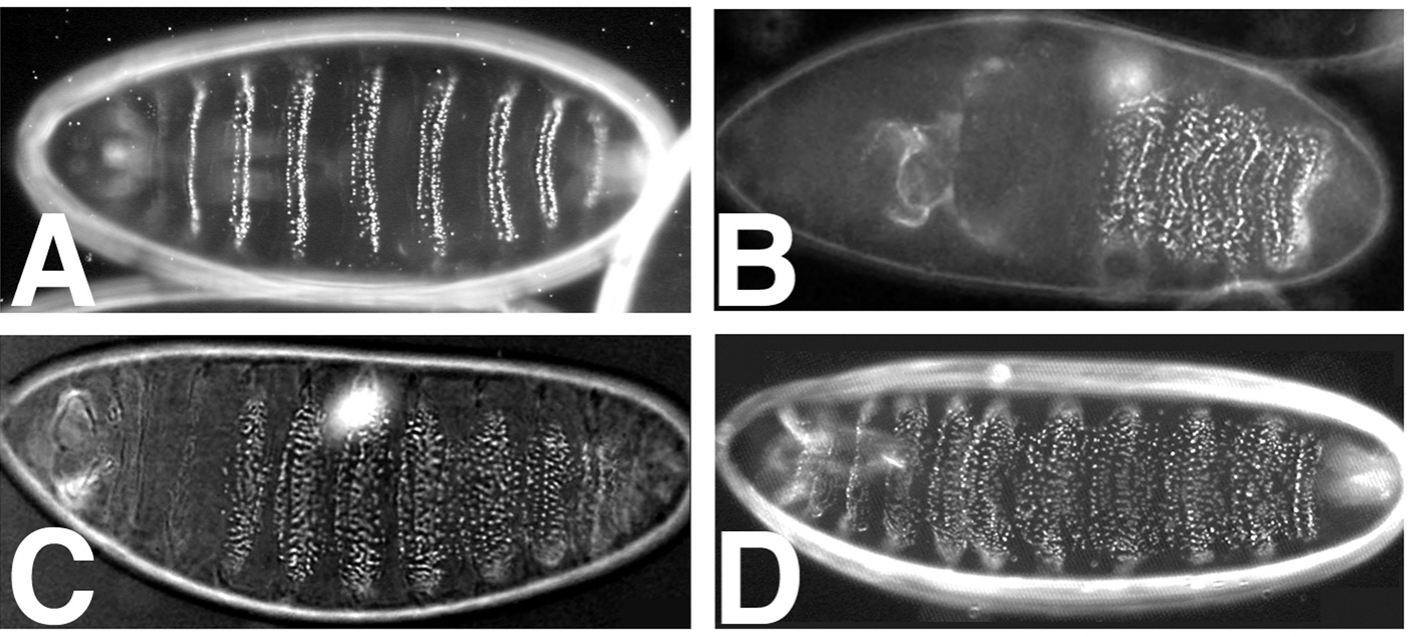
**Rescue potential of Arm^Δ*N*-88^**. Larval cuticles were prepared from wildtype (A), *arm*^*YD*35 ^hemizygous (B), *arm*^*YD*35 ^expressing wildtype Arm (C) or *arm*^*YD*35 ^expressing an N-terminal deletion of Arm, Arm^Δ*N*-88 ^(D). Ectopic expression was achieved using the UAS/Gal4 system and *arm::Gal4 *as a driver line. Note that *arm*^*YD*35 ^has a strongly shortened cuticle lacking anterior and head structures. Wildtype Arm and Arm^Δ*N*-88 ^are able to partially suppress both the segmentation defect and cuticle formation.

How does cleavage of Arm trigger destabilization of cell adhesion in apoptotic epithelial cells? One possibility is that cleavage of Arm results in a release of *D*E-cadherin from the plasma membrane and therefore is not active in cell adhesion. To test the function of the N-terminally truncated Arm we generated a transgene in which the N-terminus of Arm was deleted up to D88, called Arm^Δ*N*-88^. The adhesive function was tested in a genetic assay using the Gal4-UAS system to rescue the zygotic phenotype of a strong loss of function allele, *arm*^*YD*35^. The *arm *loss of function phenotype was partially rescued by expression of wildtype Arm and by the Arm^Δ*N*-88 ^construct to a similar extent (Fig. 7). In addition to a partial rescue of the segmentation defect, adhesion defects of zygotic *arm*^*YD*35 ^hemizygotes, reflected by the lack of anterior structures like anterior cuticle, head segments and cephalopharyngeal skeleton were rescued by expression of Arm^Δ*N*-88 ^(Fig. 7B, C, D). These data suggest that N-terminal truncation of Arm at D88 does not completely abolish the adhesive function of the protein. Therefore Arm cleavage during apoptosis is likely to not just render the protein functionally inactive suggesting that other mechanisms contribute to the breakdown of cell adhesion in apoptotic cells.

## Discussion

Apoptosis represents an irreversible cellular differentiation program that results in the formation of metabolically inactive apoptotic vesicles to be eliminated by phagocytic cells. While the molecular pathways that control the execution of cell death as well as the activation and the activity of caspases have been studied in great detail, the events downstream of the execution, namely the function of individual target cleavage by caspases, are just beginning to emerge. The central question in this field is how caspase cleavage alters the activity of the proteins to orchestrate the changes in the morphology and the physiology of the dying cell. The cellular differentiation triggered by caspases might also be of interest defining the emerging role of caspases in processes unrelated to apoptosis like cell communication and differentiation [38-40].

The present study demonstrates that cell death in DIAP1 mutant *Drosophila *embryos represents a well-defined model system to study the specificity and function of caspase targets in apoptotic cells. DCP1 and drICE are activated in DIAP1 mutant embryos and DIAP1-dependent cell death in the early embryo requires the activity of drICE and DCP1 [24,28,41,42]. The activation of caspases in DIAP1 deficient embryos depends on the Apaf-1 homolog Dark and the CARD-domain caspase DRONC [43,44]. Dronc and Dark are required for most cell death throughout the *Drosophila *life cycle as only little cell death occurs in loss-of-function mutants for the respective genes [44-48]. The requirement of Dark and Dronc for DIAP1-dependent cell death indicates that cell death in DIAP1 deficient embryos is under similar control as naturally occurring cell death.

drICE and DCP-1 are the closest relatives to caspase 3, the major effector caspase in vertebrates. Although DCP1 is highly related to drICE and exhibits very similar substrate specificity, Arm is cleaved by drICE, but not DCP1 in vitro. These results suggest that DCP1 and drICE, despite all similarities described so far, have distinct substrate specificities. Consistent with this conclusion, DCP1 and drICE exhibit both overlapping and differential functions: While some cells in the embryo require both DCP1 and drICE for apoptosis, some cell types require either of these caspases [41]. The cleavage product of Arm obtained by drICE in vitro has the same apparent molecular weight as the cleavage product detected in apoptotic cells in embryo extracts of DIAP1 mutants. Mutation of the DQVD^88 ^motif to DQVA^88 ^renders the protein resistant to cleavage both in vitro and in vivo, providing strong evidence that in apoptotic cells Arm is cleaved at DQVD^88^. In conjunction with the observations that drICE is highly expressed in the early embryo, is activated in DIAP1 mutant embryos and is required for cell death in DIAP1 mutant embryos, the results demonstrate that Arm is specifically cleaved by drICE in *Drosophila *embryonic apoptotic cells.

The DQVD motif is conserved in insects but it is not in vertebrate homologs of beta-catenin. Strikingly however an important caspase cleavage site has been determined in mammalian cells at a similar position (ADID93) suggesting that although the sequence has not been conserved, the position of the caspase cleavage site within the N-terminal domain of the protein has been conserved [32]. Moreover sequence analysis of beta-catenin throughout the animal kingdom revealed predicted caspase cleavage sites at very similar positions within the N-terminal domain of beta-catenin homologs in fish, cephalochordata, annelida and nemertea (Müller, unpublished data). These observations support the idea that N-terminal truncation of beta-catenin by caspases might represent an important mechanism in morphological changes that occur during apoptosis of epithelial cells.

In cultured epithelial cells, E-cadherin is subject to extra- and intracellular degradation after induction of apoptosis [49]. Our results suggest that removal of the N-terminal peptide does not abolish the adhesive function of Arm. This result is not unexpected as the *D*E-cadherin as well as *D*α-catenin binding domains of Arm are not affected by drICE cleavage. Previous studies in mammalian cells are consistent with our data in that the central Arm repeats region remains unaffected by caspase cleavage. Moreover, when anoikis is induced in human breast epithelial cell lines or MDCK cells, only beta-catenin is degraded, while α-catenin and E-cadherin remained uncleaved [4,32,50,51]. In this case, the truncated forms of beta-catenin were still able to bind E-cadherin and α-catenin, but still might not be able to compensate for the normal function of full-length beta-catenin in cell junctions. It is interesting to note in this context that N-terminally truncated Arm when localized in the cytoplasm is constitutively active for Wg/Wnt signalling, since it lacks phosphorylation sites for Zeste White-3 Kinase and Casein Kinase 2 [52,53]. Since Wg signalling is required for cell survival, activated Arm in the cytoplasm might suppress apoptosis; this mechanism provides an alternative explanation for the rescue potential of Arm^Δ*N*-88 ^[53].

Does cleavage of the N-terminus of Arm by drICE contribute to the destabilisation of adherens junctions during apoptosis? To directly address this question we asked whether cleavage by drICE results in inactivation of the adhesive function of Arm by over expressing drICE resistant Arm in DIAP1 mutant embryos. Although Arm^D88A ^expression delayed apoptosis, expression of wild-type Arm delayed the onset of apoptosis to a similar degree and thus this experiment did not reveal a specific function of Arm cleavage by caspases (Kessler and Müller, unpublished results). Therefore it is not possible to determine directly whether cleavage by drICE leads to inactivation of Arm, because over expression of a caspase substrate per se might quench caspase activity resulting in a delay of the onset of apoptosis.

One potential effect of Arm cleavage could be the release of an N-terminal fragment that might actively interfere with the stability of the cadherin-catenin complex in adherens junctions. For example it has been shown in a human tissue culture system that the N-terminal domain of beta-catenin can bind to the central region of beta-catenin and might thereby interfere with its binding to cadherins [54]. However our biochemical analyses failed to provide evidence for the presence of such a stable N-terminal fragment in apoptotic cells. An alternative possibility is that drICE-mediated cleavage of Arm results in a reduction of its binding properties to *D*E-cadherin. This model would also explain the delay in the removal of *D*E-cadherin from the plasma membrane upon expression of the drICE-resistant form of Arm and is also consistent with a recent report showing that *D*E-cadherin is delocalised in DIAP1 mutant embryos [27]. Most notably, this study also suggested that cleavage of Arm might act upstream of the down-regulation of the cadherin-catenin complex during apoptosis. The data presented here suggest that cleavage of the Arm N-terminal domain by drICE leads to a disruption of its interaction with *D*E-cadherin and/or with other yet to be defined components of the adherens junctions that are affecting the association of the cadherin-catenin complex in apoptotic cells. For example, deletion of the N-terminus might impair the capability of *D*E-cadherin to bind to the central Arm repeats as shown for human beta-catenin [54]. It will therefore be interesting to define the nature of this potential intra-molecular interaction and to determine whether the N-terminus binds additional regulatory proteins that are involved in the adhesive properties of beta-catenin in epithelial cells.

## Conclusion

Our work addresses the question which caspase targets trigger changes in cell adhesion and adherens junctions when polarized epithelial cells undergo apoptosis. We demonstrate that beta-catenin (Armadillo) is a direct target of the caspase drICE in *Drosophila *epithelial apoptotic cells. Cleavage of Arm by drICE is specific for a single caspase consensus site located in the amino-terminal domain of the protein. Mutation of this site results in a caspase-resistant form of Arm, which remains intact when expressed in apoptotic cells. Expression of caspase-resistant Arm in apoptotic cells delays disassembly of *D*E-cadherin from cell junctions. We therefore conclude that cleavage of Arm is an important event for dismantling of epithelial cells during apoptosis. This study also highlights that functional analyses of specific caspase target cleavage events in apoptosis can be complicated because of the complexity of proteolytic cleavages by caspases required to disassemble large protein complexes such as adherens junctions. The apoptosis model of DIAP1 deficient embryos that was used in this study provides the opportunity of a systems approach to caspase cleavage that might be able to explain the biochemical mechanisms responsible for this terminal differentiation process.

## Methods

### Molecular cloning and site directed mutagenesis

Molecular cloning was performed after standard protocols. Arm^D88A ^was generated by site directed mutagenesis of the Arm cDNA in the pBluescript KS+ vector using the primer ArmD88A: 5' CAAGACCAAGTGGCT-GATATGAACCAG and its reverse complement changing aspartate 88 to alanine. The other mutated forms of Arm were generated using the primers Arm^D123A ^(CCACCCAGTTTGCGCCCCAACAGCCG); Arm^D170A ^(CAAGCTGCTGAACGCGGAGGATCAGGTGG), Arm^D172A ^(TGCTGAA-CGATGAGGCGCAGGTGGTAGTAG) and Arm^D755A ^(CTCTACCAATAGCGTCGA-TGCAGGGTCTG). Wildtype and mutated Arm cDNAs were used for in vitro caspase cleavage experiments. All mutations were confirmed by sequencing. Arm^D88A ^was also used to generate transgenic flies carrying UAS::Arm^D88A^. The mutated Arm cDNA was cloned in frame to a modified pUAST vector carrying an N-terminal FLAG epitope using Not1 and Xba1 introduced with the primer pair 5' AAGGAAAAAAGC-GGCCGCCACCATGAGTTACATGCCAGCCCAGAAT3' and 5'GCTCTAGACTAACAATCGGTATCGTACCAGG3'.

### Generation of the Arm^Central ^Antibody

The central domain of Arm containing the Arm repeats as well as a short part of the C-terminus was cloned from the Arm cDNA into the pQE30 vector with BamH1 and Pst1 after PCR using the primers 5' CGGGATCCATGAGTTACATGCCAGCCCAGA-AT 3' and 5' AACTGCAGCTAACAATCGGTATC-GTACCAGG 3'. Expression of the protein was carried out in E. coli M15 cells for 4 h at 28°C with a final concentration of 1 mM IPTG. The protein was purified with Ni^2+^NTA matrix under denaturing conditions in 6 M GuHCL, eluted and refolded in ice-cold buffer containing 50 mM HEPES pH 7.5, 200 mM NaCl, 1 mM DTT and 1 M NBSB-201. The eluted protein was used for immunization of a rabbit using standard protocols. Recombinant Arm^Central ^protein was immobilized using the amino-link plus kit (Pierce) and used for affinity-purification of Arm^Central ^antibodies. Arm^Central ^antibodies were characterized by Western blotting of embryo protein extracts and immunofluorescence staining of embryos. To verify that Arm^Central ^antibodies do recognize Arm from embryo protein extracts, *D*E-cadherin/catenin complexes were immunoprecipitated using the mouse monoclonal Arm^Nterm ^7A1, rabbit anti Arm^Central ^and rat anti *D*E-cadherin antibodies and subsequent western blotting using 7A1 and Arm^Central ^antibodies. Embryo lysates were prepared through grinding embryos in ice-cold CHAPS buffer (0,2% CHAPS, 10 mM Di-thiotreitol (DTT), 100 mM HEPES, 200 mM NaCl, 20% Sucrose). Actin was used as a standard, because it represents an abundant protein in early embryos. We found that actin appears to be stable in *th*^109 ^apoptotic cells.

### Immunohistology

For immunofluorescence, embryos were fixed either by the heat/methanol method [22] or with 4% Paraformaldehyd in PBS (in case of TUNEL staining). Primary antibodies used for immunohistology were: mouse-anti-Arm^Nterm ^7A1 (DSHB 1:10), rat-anti-*D*E-cadherin (DSHB 1:20), mouse anti Neurotactin (DSHB 1:10), rat anti *Dα*-catenin (1:20), rabbit anti Arm^Central ^(1:250), mouse anti Flag M5 (Sigma, 1:1000), mouse anti En (DSHB 1:10) and rabbit anti beta-galactosidase (1:5000) or mouse anti beta-galactosidase (DSHB 1:100). TUNEL staining was performed as described before using the in situ cell death detection kit (Roche, Mannheim;[24]).

### *In vitro *cleavage of Arm

^35^S-labeled Arm protein was in vitro translated using the T7/T3 rabbit reticulocyte lysate system (Promega) using cDNAs of full-length or Arm variants, respectively. Extracts of wildtype or *th*^109^/TM3[*ftz*::*lacZ] *embryos were made under conditions previously shown to keep caspases active in these extracts [24]. ^35^S-labelled Arm proteins were incubated with 30 μg of extracted protein for 4 h on 30°C. The reaction was stopped though boiling in SDS sample buffer and the sample was subjected to SDS PAGE following autoradiography. Cleavage of the Arm protein was blocked by addition of the pan-caspase inhibitor z-VAD-FMK at 20 nmol [31]. For experiments with the recombinant proteins, His-tagged versions of drICE, DCP1 and Decay were induced in BL21pLysS bacteria and purified under native conditions as described [24]. Plasmids were a generous gift of Soon Yi Yoo and Bruce Hay (Caltech, USA). Various amounts of caspases were used for cleavage of Arm as indicated. For all experiments in vitro translated Lamin Dmo protein was used as a positive control for active caspases in the extracts and in the purified recombinant caspase fractions. Cleavage of Lamin Dmo protein by caspases has been described before [30]. The plasmid encoding *Drosophila *Lamin Dmo was a kind gift of Paul Fischer (Stony Brook, USA).

### Drosophila genetics

Fly work and embryo collections were performed after standard protocols. Apoptotic cells were recorded in embryos homozygous for the DIAP1 null allele *th*^109^, which carries a nonsense mutation in the N-terminus resulting in a protein lacking all known functional domains [26]. As controls, Oregon R or *w*^1118 ^embryos were examined. Transgenic lines carrying a *UAS::Arm*^*D88A *^transgene were obtained by standard methods. A 2nd chromosomal insertion was further used to obtain *UAS::Arm*^*D88A*^/*UAS::Arm*^*D88A*^; *th*^109^/*TM3 [ftz::lacZ]*. Males of this stock were crossed to females of the recombinant *th*^109^*mat15::Gal4*/*TM3 [ftz::lacZ] *stock; this cross allows expression of the UAS transgene with onset of zygotic gene expression in the early embryo.

## Authors' contributions

HAJM and TK conceived the project. TK designed and performed the experiments, prepared the figures and assisted in drafting the manuscript. HAJM planned and participated in the design of the experiments and performed some of the experiments. HAJM drafted and edited the manuscript. Both authors read and agreed on the final draft.
